# Psychological Needs, Satisfaction and Intention to Migrate in Iranian Nurses: A Qualitative Content Analysis

**Published:** 2018-08

**Authors:** Vahid SHOJAEIMOTLAGH, Sousan VALIZADEH, Hadi HASANKHANI, Arezoo BOZORGOMID

**Affiliations:** 1. Dept. of Medical Surgical Nursing, Faculty of Nursing and Midwifery, Tabriz University of Medical Sciences, Tabriz, Iran; 2. Dept. of Pediatrics, Faculty of Nursing and Midwifery, Tabriz University of Medical Sciences, Tabriz, Iran; 3. Asadabad School of Medical Sciences, Asadabad, Iran

**Keywords:** International migration of nurses, Psychological needs, Job satisfaction, Content analysis

## Abstract

**Background:**

The shortage of nurses is a global health problem and one of the main challenges for healthcare systems throughout the world including Iran. One of factors that affect the migration trends is psychological needs satisfaction. This study aimed at identifying the psychological factors which persuade Iranian nurses to migrate and suggesting necessary measures in this regard.

**Methods:**

This qualitative study was conducted through a traditional content analysis approach on 20 working Iranian nurses who are migrating to other countries between 2015 and 2017. Data were collected through interviews and observation in different wards of hospitals in Iran. Data were written and analyzed after reduction, naming them, obtaining analytical codes, and identifying the categories and subcategories using traditional method.

**Results:**

Three main categories in relation to satisfaction of the psychological needs and reasons for migration were obtained after data analysis. The first category was “authority” with two subcategories of independent decision-making power and being unconsidered, the second category was “social support” with three subcategories of communication with health team members, communication with nurse colleagues, and communication with nurse managers, and the third category was “job promotion” with four subcategories of addressing the routines, nurse role, job promotion opportunities, and teaching organizational environment.

**Conclusion:**

Identifying problems and obstacles to achieve the goal is the first step to solve the problem. This study provided further and clearer understanding of psychological causes regarding decision of nurses to migrate to developed countries, and nurses noted that the decision to migrate is in the search to meet their psychological needs.

## Introduction

Manpower is the most effective element in the survival and success of any organization. Enjoying of capable human capital is a major advantage in today’s organizations in which permanent changing and impact from environment are accepted as an inevitable necessity (1). Nursing staff shortage is a global problem (2, 3). Shortage of nurses and other healthcare workers is referred as the human resources crisis in health, and nurse shortage is a challenge in a number of industrialized and advanced countries (4). The shortage is usually described and measured at the levels of country staff, resources, and demand estimate for health services, therefore, it cannot be easily quantified. Thus, the lack of nursing staff is a multidimensional phenomenon (5).

There are more than 20.7 million nurses and midwifes around the world. WHO has estimated a shortage of almost 9 million nurses and midwives throughout the world. Although the nursing profession has been faced with such a problem in the past also, this now becomes one of the main challenges of the health care system in the world (6). Shortage of nurses in the healthcare system affects disease prevention, health promotion, satisfaction from nursing service and nursing services quality, so that the shortage leads to mandatory overtime of nurses and consequently to fatigue and burnout, increased nursing errors, reduced nursing services quality, and in a vicious cycle, to job leave (7–13).

Several factors are involved in the development of nursing staff shortage in the world, among them the followings can be pointed out: poor distribution of nurses in the world, lack of correlation between the policy objectives of education and health, unsuitable working environment, lack of evidence for policy and decision-making, reduced university enrollment, burnout and subsequent leaving, increased demand for health care, immigration and lack of professional identity of incorrect nursing image. In addition to the listed issues, a number of socioeconomic trends including increased aged population, advanced technology, and increased health expectations regularly affect health care system and increase the growing need for experienced nurses (13–18).

Migration of educated nurses to the developed countries is a factor that exacerbates the nursing shortage in Iran. According to a member of the Supreme Council of the Iranian Nursing Organization, about 1000 nurses annually migrate abroad through employment channels.

Mental health or psychological factors are among the factors that exacerbate the flood of immigration (19). Mental health refers to the feeling of well-being and ensuring of self-effectiveness, self-reliance, capacity of competition, and self-actualization of intellectual and emotional potential which enable the person to perform the tasks in the workplace through coordinating with others (20). Mental health is of utmost importance since it is directly related to individual-social performance and social damage. Undesirable mental health is associated with serious damages in job performance and this damage includes relevant tasks and interpersonal and transpersonal aspects. Mental disorders, medication errors, patient safety, job motivation, job satisfaction, and job leave are all in relation to mental health (19), therefore, paying attention to this issue and identification of factors which guarantee a part of the phenomenon not only may have a direct impact on the performance of nurses, but also in its scope, can prevent job leave or migration of nurses to other developed countries to achieve mental health and other better items.

This study was derived from a qualitative thesis through the Grounded theory in which the psychological factors related to migration were investigated in Iranian nurses, and as the main question of this study, the psychological factors involved in decision of nurses for professional migration were identified.

## Methods

This qualitative study aimed to identify and explore reasons for the decision to migrate in Iranian nurses working in hospitals of medical sciences universities in terms of psychological factors with traditional content analysis method.

### Data collection

The basic data were collected through the goal-oriented and snowball sampling methods, and theoretical sampling was performed after the advent of codes and primary categories and continued until data saturation. The data were collected at baseline using semi-structured interviews. A few general questions about nursing, the ward, and some demographic questions were asked to gain confidence in participation. The interview was continued with the general question “tell when did you decide to migrate?” and semi-structured questions including questions such as “why do you want to migrate?” “What are your problems persuading you to migrate?” and “what caused you to be tired to work here?” Interviews took between 30 to 90 min. The length and place of the interview were chosen by participants.

### Participants

Twenty-one undergraduate and graduate nurses (13 men and 7 women) consisting of 2 educational supervisors, 1 clinical supervisor, 2 head nurse and 15 experienced nurses who were working in hospitals of medical sciences universities and were introduced by the Iranian Nursing Organization to the researcher between 2015 and 2017 participated in this study. Their mean work experience was 9 yr, and their age range was 24 to 45 yr with an average of 33 yr. At first, the selection criteria were willingness to participate in the study and express their experiences, passing of one year after proceeding for immigration and referring to the Nursing Organization to receive the certificate of professional competence, and availability for interview. However, these were changed later according to theoretical sampling. Age, gender, work experience, degree of education, etc. were considered in order to take account the maximum diversity of participants.

### Data analysis

First, all recorded interviews were written and reviewed. The first interview was coded and analyzed using MAXQDA. In order to extract the original codes, the texts obtained from interviews and observations were read word by word several times to achieve a general perception from the data and to specify words or phrases that represent ideas or concepts. The codes were named by continuation of this process, and based on similarities and differences; they were divided into subcategories which were more abstract than the initial codes. In order to create the main categories, the subcategories extracted in the previous step were combined according to the relationships, differences, and similarities. At the same time with data analysis, the process of data collection continued until data saturation, i.e. as long as interviews did not give any new information and the entries were repeated. The main categories were finally revealed.

### Study precision

Validity and reliability of this study were tested using the criteria proposed by Guba and Lincoln. To test credibility, the author had a long-term cooperation and involvement with the participants. Several reviews were conducted by supervisors and their comments were used in this field. In addition, five participants confirmed the compatibility of the findings with their clinical experiences; and in order to increase the dependability, the text review was limited at baseline to prevent bias in the study. To increase confirmability, the research steps and the research process were clearly recorded and reported; and to increase transferability, the maximum diversity of participants was used.

### Ethical aspects

The study was launched after permission from the Ethics Committee of Tabriz University of Medical Sciences and obtaining a written certificate from the University. The participants were asked to fill out a written consent, and all their information will remain confidential and no name will be declared.

## Results

The data from interviews, notes in the field, and reminders were analyzed and a total of 122 open codes in 8 subcategories and 3 categories were obtained in this context. The main categories were authority, emotional support, and job promotion, each with subcategories described below (Table 1).

**Table 1:** Categories and subcategories related to the concept of inner enrichment or satisfaction

***Category***	***Subcategory***
Authority	Independent decision-making power
	Being unconsidered
Social support	Communication with health team members
	Communication with nurse colleagues
	Communication with managers (head nurse, supervisor, matron)
Job promotion	Addressing the routines
	Nurse role
	Job promotion opportunities

### Category 1: Authority

Authority was introduced by the participants as one of the components of inner enrichment supplier. This concept was also confirmed by them. Before completion of categories in theoretical sampling, the participants were asked to define the authority by own words and express representative instances of authority. In all instances mentioned, the words power and authority were somehow used.

#### 1.1 Independent decision-making power

The participants introduced nursing as the most dependent career in the world and denied the presence of independent and even semi-independent role of nursing in Iran. Even in the roles where there are no possible complications, they stated that nursing is totally dependent and they cannot do anything by their own judgment and decision.

Participant 1 (nurse, male, 28 yr old) said: “Nursing process is a concept which does not exist in Iran’s nursing, let alone that you diagnose, plan, and implement it as nursing in various stages. It is really very funny incident to speak of nursing process. Though, if someone wants to run it, is it possible with a lot of patients cared for only one nurse?”

#### 1.2 Being unconsidered

The nurses knew themselves out of all clinical and non-clinical decisions and believe that nurses are outside the circle of decision-making in any situation. When the nurses were asked about teamwork, most of them laughed and said, what is teamwork, do we have and is it possible.

Participant 3 (nurse, female, 34 yr old) said: “You do not know but believe me. They want to mend your workplace, but they do not ask your opinion. For example, they want to put a shelf for drugs with which I’m constantly dealing. My opinion should be important for them, but it isn’t.”

Clinical decision is the main part of professional performance of nurses and can distinguish professional nurses from unprofessional care staff. Clinical decision influences the quality of care more than anything and helps identify the needs of patients determine the best nursing action.

#### Category 2: Social support

Supportive work environments are a major cause of job satisfaction for nurses, and enjoying social support in nursing affects patients care, job satisfaction, and attraction and retaining of manpower in the organization. Many studies have shown that the effectiveness of nurses increases when they have strong social support. An environment with high social support reduces job stress and retains nurses in the organization and finally compensates for the shortage of nurses, however, perceived social support resources are considered very inadequate by nurses.

#### 2.1 Communication with health team members

According to most nurses, members of health groups and on top of them, doctors are on the contrary of nurses and an ordering relationship exists between nurses and doctors. Paternalism, dictatorship, and wasters of nurses’ rights are words that were attributed by nurses to physicians.

In this regard, participant 17 (head nurse, male, 41 yr old) said: “there is no cooperation between us and the doctors and somehow we are their subaltern. We are always guilty and if we encounter a problem, not only doctors do not support us, but the system which is a physician governor-ship system supports the doctors.”

#### 2.2 Communication with nurse colleagues

The interaction between nurse colleagues and the relationship between them was not in a good condition. Nurses noted that the colleagues do not understand them in many cases and in cases where a person is in trouble for different reasons, they not only do not understand him but also pull rug from under him and in a competitive situation, try to exaggerate the defects of others and show their superiority.

In this context, participant 8 (nurse, female, 29 yr old) said: “Jealousy is filled among co-workers, especially among women to show themselves.” Another participant (training supervisor, female, 34 yr old) said: “I never forget. I was in the staffing plan and after two months I was relatively weak in venipuncture. I had a friendly relationship with the head nurse but one of my colleagues constantly talked with the head nurse that I deteriorate the veins.”

#### 2.3 Communication with managers

Poor communication of nurse with head nurses and supervisors, ordering relationship, catch red-handed, retaliation, lack of cooperation for leave, view from the top, distrust, disrespect, grandiosity were the words which described the relationship between nurses and managers.

In this regard, participant 12 (nurse, female, 24 yr old) said: “When I was night shift and wanted to deliver the shift at morning, the head nurse always brings me to tears and nags indiscriminately during shift delivery.”

### Category 3: Job promotion

Nursing staff promotion is a current, dynamic, and unique notion that can be explained based on the work environment and interpersonal interactions. This social process forms in interacting with people and existing situations in the work field and environment; according to the nurses, the formation of such a phenomenon is very weak.

#### 3.1 Addressing the routines

The concept of innovation in nursing means transforming ideas into new methods and solutions in patient care which can ultimately improve the quality of care. Therefore, in clinical care which gradually becomes routine, problem-solving skills and innovation are integrated into the other capabilities of nursing profession; however, there is any initiative and innovation in the nursing system.

Participant 18 (supervisor, female, 32 yr old) said: “According to my friend, we are wasting here. I think the things we are doing now and our approaches are about twenty yr ago and all we really have become routine. We learned a series of things and forget many things.

#### 3.2 Nurse role

Although in various countries, the role of nurses is defined in different areas of health and they can practice healthcare outside medical centers, the role of nurses is limited to patient bedside and even to a declined form in Iran. When defining the concept of authority and its role in shaping nurse satisfaction and in general, the factor influencing the nurses’ decision to migrate, the participants emphasize the words “nurse as injector,” “nurse as dressing changer,” “nurse as doctors subordinate” and in general, only to the caring roles of nurses. According to them, the role of nurse is limited only to care, and the nurses are ignored in the prevention, education, and improving public health knowledge and the lack of other roles for nurses will normally reduce their power and they are not happy in this regard.

Participant 5 (nurse, female, 36 yr old) said: “I remember I was in the first semester of nursing and studied the roles of nurses. Oh, how much they were. I told my friend that we really have many responsibilities and we should work too much; we have to be teacher, therapist, supporter, coordinator; but when I came to the hospital, I told how many books are written, while the role of the nurse is only to insert IV lines and change dressings.”

#### 3.3 Job promotion opportunities

Many participants raised the need of organization as the first priority for providing job promotion opportunities and believed that the lack of need is an obstacle for the emergence of promotion opportunities.

In this context, participant 8 (male, 35 yr old) said: “I became training supervisor after 10 yr of work, through a relationship that later I made with the matron.”

Another participant (nurse, female, 30 yr old) said: “I was accepted for the master and I wanted to continue my education, but I failed to get an educational mission despite many efforts because there are manpower shortage and low number of nurse.” Another participant said: “You cannot believe, I enrolled in a perfusionist course, but when I raised it, they wrote my shifts in the hospital that I can’t contact with my classmates and can’t attend in the classes.”

## Discussion

Human needs have been discussed by different psychological theories. These needs have been noticed at different levels. Some human needs are of particular importance for different reasons including stability over time and their profound impact on behavior and personality. These needs, such as the need to excel, are considered psychological in nature by some researchers. Therefore, psychological empowerment can serve as a possible solution to increase the satisfaction of nursing staff, work motivation, and consequently the survival of the individual in the organization. Nurses in the present study were seeking to somehow satisfy their psychological needs. Although satisfaction of psychological needs is not all reasons for migration of nurses, it is the important and notable cause of nurses’ immigration which is different from all the reasons mentioned in studies. Nurses are willing to change their workplace in order to satisfy their psychological needs. They want to have the authority, be supported and have job promotion opportunities.

### Theme number one: Authority

The lack of work independence and decision-making power as well as not participating in decision making or in other words “being unconsidered” are the factors of job dissatisfaction cited by the nurses in this study; these factors persuade them to change their workplace in order to achieve job satisfaction. The participation of people in decision-making helps to value them and their work increases their intrinsic motivation and empowerment (21) and affects job satisfaction, commitment, responsibility, productivity, and quality of service (22). On the other side, increased capability reduces the employees’ turnover, fatigue, and absenteeism, and in the shadow of intrinsic motivation, people will be more committed to their jobs (23, 24). As the nurses in this study decided to migrate in the hope of a better workplace and to rebuild their damaged self-esteem due to lack of respect and value for decision-making. Other research reported significant relationships between psychological empowerment and affective commitment, job satisfaction, and organizational commitment which are factors effective on the attitudes and motivation of staff to the job (23, 24).

### Theme number two: social protection

Supportive work environment is the main factor of job satisfaction for nurses, and enjoying of social support in nurses affects patients care, employees’ job satisfaction, and attraction and retaining of manpower in the organization (25, 26). When nurses have strong social support, job stress is declined and nurses are retained in the organization and finally may compensate for the shortage of nurses (25). Thus, the perceived social support is an important inhibiting factor of negative effects of job stress, one of them is decision to migration in the present study. Support and trust of colleagues indicate the social support and is one of the reasons for nurses to continue to work despite the un-ideality of other factors. In the present study, nurses have not adequate social support from different people of the organization.

Less interaction between nurses with each other and nurses with nursing managers, result in less social support of nurses and this, in turn, shadows other workplace problems (27). Therefore, support of authorities can be a cause of satisfaction and reduced job stress in people, nursing officials should understand the nature of social networks and its impact on improvement of job satisfaction of nurses and maintain the organization and prevent decision of nurses to migrate.

### Theme number three: Job promotion

Psychological empowerment and structural capacity both can do help in job satisfaction. Therefore, nurses are looking to find work environment which improves both of these concepts to ultimately yields to job satisfaction (27, 28). The motivation for job satisfaction is so high that nurses are prepared to bear immigration problems in this study. The rate of retaining of nurses in the nursing profession is directly associated with the psychological and structural empowerment (28, 29).

Job enrichment results in provision of responsibilities and more employment challenges for the staff. Job enrichment can provide employees to have the authority to make decisions in their work. Job enrichment and its aspects, i.e. multiple roles or role development and promotion and career advancement opportunities can increase job satisfaction and motivation and reduce employee absenteeism (28, 30). In this study, routine and repetitive tasks were boring for nurses and destroyed their creativity and innovation.

## Conclusion

Nurses’ job satisfaction is affected by multiple components, psychological needs are one of them which includes formal and informal authority, social support, and job promotion and empowerment opportunities. Failure to meet them persuades nurses to seek for a new work environment and since work environment seems to be the same in Iran, they decide to migrate to developed countries to improve the quality of their work life. Therefore, health policy-makers can use the results of this study and provide job satisfaction of nurses in the field of psychological needs and turn at least one of the facilitators of decision of nurses to migrate to obstacles of decision to migrate.

## Ethical considerations

Ethical issues (Including plagiarism, informed consent, misconduct, data fabrication and/or falsification, double publication and/or submission, redundancy, etc.) have been completely observed by the authors.

## References

[1] Salami B Dada FO Adelakun FE (2016). Human Resources for Health Challenges in Nigeria and Nurse Migration. Policy Polit Nurs Pract, 17:76–84. 27365339 10.1177/1527154416656942

[2] Booth RZ (2002). The nursing shortage: a worldwide problem. Rev Lat Am Enfermagem, 10:392–400. 12817393 10.1590/s0104-11692002000300013

[3] Hudspeth R (2013). Staffing healthy workplaces: some global nursing shortage issues. Nurs Adm Q, 37:374–6. 24022291 10.1097/NAQ.0b013e3182a2fa65

[4] Hawkes M Kolenko M Shockness M Diwaker K (2009). Nursing brain drain from India. Hum Resour Health, 7:5. 19187559 10.1186/1478-4491-7-5 PMC2642753

[5] MacLean L Hassmiller S Shaffer F (2014). Scale, Causes, and Implications of the Primary Care Nursing Shortage. Annu Rev Public Health, 35:443–457. 24422561 10.1146/annurev-publhealth-032013-182508

[6] Alliance GHW (2015). Health workforce 2030 – towards a global strategy on human resources for health. http://www.irna.ir/en/News/2716240/Social/Iran%E2%80%99s_per_capita_milk_consumption

[7] Naicker S Plange-Rhule J Tutt RC Eastwood JB (2009). Shortage of Healthcare Workers in Developing Countries-Africa. Ethn Dis, 19(1 Suppl 1):S1-60-4. 19484878

[8] Ross SJ Polsky D Sochalski J (2005). Nursing shortages and international nurse migration. Int Nurs Rev, 52:253–62. 16238721 10.1111/j.1466-7657.2005.00430.x

[9] Geun HG Redman RW McCullagh MC (2016). Turnover and Associated Factors in Asian Foreign-Educated Nurses. J Nurs Adm, 46:271–277. 27093183 10.1097/NNA.0000000000000342

[10] Schmiedeknecht K Perera M Schell E (2015). Predictors of Workforce Retention Among Malawian Nurse Graduates of a Scholarship Program: A Mixed-Methods Study. Glob Health Sci Pract, 3:85–96. 25745122 10.9745/GHSP-D-14-00170 PMC4356277

[11] Buchan J O’May F Dussault G (2013). Nursing Workforce Policy and the Economic Crisis: A Global Overview. J Nurs Scholarsh, 45:298–307. 23656542 10.1111/jnu.12028

[12] Buchan J Twigg D Dussault G Duffield C Stone PW (2015). Policies to sustain the nursing workforce: an international perspective. Int Nurs Rev, 62:162–170. 25639942 10.1111/inr.12169

[13] Nantsupawat A Kunaviktikul W Nantsupawat R (2017). Effects of nurse work environment on job dissatisfaction, burnout, intention to leave. Int Nurs Rev, 64:91–98 27882573 10.1111/inr.12342

[14] Green A Kishi A Esperat MC (2010). State policy and research initiatives focused on improving nursing workforce: an integrative literature review. Annu Rev Nurs Res, 28:63–112. 21639024 10.1891/0739-6686.28.63

[15] Janiszewski Goodin H (2003). The nursing shortage in the United States of America: an integrative review of the literature. J Adv Nurs, 43:335–43. 12887349 10.1046/j.1365-2648.2003.02722_1.x

[16] Carriere BK Muise M Cummings G Newburn-Cook C (2009). Healthcare succession planning: an integrative review. J Nurs Adm, 39:548–55. 19955970 10.1097/NNA.0b013e3181c18010

[17] Abhicharttibutra K Kunaviktikul W Turale S (2017). Analysis of a government policy to address nursing shortage and nursing education quality. Int Nurs Rev, 64:22–32 27046433 10.1111/inr.12257

[18] [No authors listed] (2014). Nursing shortage ‘critical’ by 2017. Nurs N Z, 20:7. 25668854

[19] Dywili S Bonner A O’Brien L (2013). Why do nurses migrate? a review of recent literature. J Nurs Manag, 21:511–520. 23409815 10.1111/j.1365-2834.2011.01318.x

[20] Sato F Hayakawa K Kamide K (2016). Investigation of mental health in Indonesian health workers immigrating to Japan under the Economic Partnership Agreement. Nurs Health Sci, 18:342–349. 26875749 10.1111/nhs.12275

[21] Tingle J (2012). Perspectives on shared decision making in health care. Br J Nurs, 21:1042–3. 23123752 10.12968/bjon.2012.21.17.1042

[22] Burkett LS (2016). Collaborative decision making: Empowering nurse leaders. Nurs Manage, 47:7–10. 10.1097/01.NUMA.0000491131.60730.d3 27570916

[23] Xia Y Zhang L Zhao N (2016). Impact of Participation in Decision Making on Job Satisfaction: An Organizational Communication Perspective. Span J Psychol, 19:E58. 27644126 10.1017/sjp.2016.56

[24] MacPhee M Wardrop A Campbell C (2010). Transforming work place relationships through shared decision making. J Nurs Manag, 18:1016–26. 21073573 10.1111/j.1365-2834.2010.01122.x

[25] Goh YS Lopez V (2016). Job satisfaction, work environment and intention to leave among migrant nurses working in a publicly funded tertiary hospital. J Nurs Manag, 24:893–901. 27169747 10.1111/jonm.12395

[26] Boamah SA Read EA Spence Laschinger HK (2017). Factors influencing new graduate nurse burnout development, job satisfaction, and patient care quality: A time-lagged study. J Adv Nurs, 73:1182–1195. 27878844 10.1111/jan.13215

[27] AbuAlRub R El-Jardali F Jamal D AbuAl-Rub N (2016). Exploring the relationship between work environment, job satisfaction, and intent to stay of Jordanian nurses in underserved areas. Appl Nurs Res, 31:19–23. 27397813 10.1016/j.apnr.2015.11.014

[28] Cicolini G Comparcini D Simonetti V (2014). Workplace empowerment and nurses’ job satisfaction: a systematic literature review. J Nurs Manag, 22:855–71. 25298049 10.1111/jonm.12028

[29] Choi SL Goh CF Adam MB Tan OK (2016). Transformational leadership, empowerment, and job satisfaction: the mediating role of employee empowerment. Hum Resour Health, 14:73. 27903294 10.1186/s12960-016-0171-2 PMC5131441

[30] Duffield C Baldwin R Roche M Wise S (2014). Job enrichment: creating meaningful career development opportunities for nurses. J Nurs Manag, 22:697–706. 23463905 10.1111/jonm.12049

